# Geometric Aortic Remodeling and Stent-Graft Migration After TEVAR: Insights from Longitudinal 3D Analysis and Literature Review

**DOI:** 10.3390/jpm15080393

**Published:** 2025-08-21

**Authors:** Mariangela De Masi, Carine Guivier-Curien, Marine Gaudry, Alexis Jacquier, Philippe Piquet, Valérie Deplano

**Affiliations:** 1 Timone Aortic Center, Department of Vascular Surgery, Assistance Publique-Hôpitaux de Marseille, Timone Hospital, 13005 Marseille, France; marine.gaudry@ap-hm.fr (M.G.); philippe.piquet@ap-hm.fr (P.P.); 2 CNRS, Centrale Marseille, Institut de Recherche sur les Phénomènes Hors Equilibre, Aix Marseille University, 13013 Marseille, France; carine.guivier@univ-amu.fr (C.G.-C.);; 3 Department of Radiology, Assistance Publique-Hôpitaux de Marseille, Timone Hospital, 13005 Marseille, France; alexis.jacquier@ap-hm.fr

**Keywords:** TEVAR, late stent-graft migration, type III endoleaks, long-term follow-up, aortic remodeling, sac growing

## Abstract

**Background**: Long-term follow-up after endovascular aortic repair (TEVAR) is crucial to detect adverse aortic remodeling, even with modern stent grafts offering enhanced flexibility and durability. Conventional imaging, based on diameter measurements, may fail to identify complications such as endograft migration. **Methods**: We conducted a longitudinal 3D geometric analysis of thoracic aortic and stent-graft evolution over 10 years in a patient treated for descending thoracic aortic aneurysm (DTAA) by endovascular treatment. A three-dimensional morphological analysis (length, tortuosity, angulation, and diameter) was carried out using advanced imaging software (EndoSize, MATLAB) to track aortic geometry and stent-graft behavior over time. A focused review of the literature on stent-graft migration, its risk factors, complications, and surveillance strategies was also performed. **Results**: This case illustrates how progressive geometric remodeling—including aortic elongation and increased tortuosity—can lead to delayed stent-graft migration and late type III endoleaks, with an elevated risk of rupture. The 3D analysis revealed early morphological changes that were undetectable using standard diameter-based follow-up. These observations are consistent with published data showing higher migration rates over time, particularly in tortuous anatomies. The literature review further emphasizes the clinical relevance of geometric surveillance, given the high rates of reintervention, morbidity, and mortality associated with stent-graft migration. **Conclusions**: This study underlines the importance of personalized and geometry-based surveillance after TEVAR. Advanced morphological assessment tools provide valuable insights for the early detection of complications and tailored patient management. Their integration into routine follow-up could help optimize long-term outcomes and prevent life-threatening events such as rupture.

## 1. Introduction

Thoracic endovascular aortic repair (TEVAR) has become the preferred treatment for descending thoracic aortic aneurysms (DTAAs), offering lower morbidity and mortality compared to open surgery. However, TEVAR leaves the diseased aortic wall in place, exposing patients to progressive aortic degeneration at the aneurysm site and at the proximal and distal landing zones. The biomechanical interaction between the stent graft (SG) and the native aorta is complex and depends on the individual characteristics of each device—such as rigidity, conformability, and radial force [1]. Despite significant advances in SG design and material durability, adverse aortic remodeling remains a common issue during long-term follow-up [2]. Radiological monitoring primarily focuses on aortic diameter evolution and SG integrity to detect material defects. However, recent studies suggest that advanced geometric parameters—such as length, curvature, and tortuosity—provide a more comprehensive understanding of aortic remodeling and may allow earlier detection of device instability [3,4,5]. These geometric markers offer a dynamic and integrative assessment of aortic morphology, which can improve the early identification of patients at risk for complications. Furthermore, baseline geometric features—including excessive tortuosity, elongation, or sharp angulations—have been identified as key predictors of long-term remodeling quality and stent-graft stability, independent of the underlying aortic pathology [6,7].

In addition, advances in three-dimensional image post-processing software have made it possible to extract morphological parameters with greater reproducibility and clinical usability. Tools such as EndoSize^®^ have shown promise for the semi-automated geometric modeling of thoracic aortic anatomy [8]. These developments support the emergence of personalized, image-guided surveillance strategies aimed at identifying patients at risk of complications based on their individual anatomical evolution.

In this article, we explore the clinical significance of geometric aortic remodeling after TEVAR, focusing on its association with delayed stent-graft migration. We present a longitudinal 3D morphological analysis of a patient followed over 10 years using Computed Tomography Angiography (CTA) multi-detector 64-row scanner (REVO EVO, General Electric Healthcare, Buc, France) and advanced post-processing software (EndoSize^®^, Therenva, Rennes, France) [8].

To contextualize our findings, we also conducted a narrative review of the literature addressing stent-graft migration, its incidence, risk factors, and clinical consequences. Through this combined approach, we aim to evaluate whether the integration of geometric assessment into routine follow-up could enhance the early detection of complications and guide individualized management strategies in patients treated with TEVAR.

## 2. Longitudinal Geometric Assessment and Clinical Illustration

### 2.1. Clinical Context and Presentation

A 52-year-old male was admitted with a 73 mm descending thoracic aortic aneurysm (DTAA). A cervical debranching procedure was performed, bypassing the left subclavian artery (LSA) from the left common carotid artery using an 8 mm Uni-Graft^®^ prosthesis. This was followed by thoracic endovascular aortic repair (TEVAR) with three Medtronic Valiant stent grafts, introduced via a transfemoral approach. Initial postoperative imaging showed sac shrinkage and no endoleak at a 1-year follow-up. At 24 months, a type IIIa endoleak occurred due to the disconnection of two SG components, requiring the deployment of a bridging stent. At 132 months, the patient presented with acute aortic syndrome. CT angiography revealed a type IIIb endoleak, sac enlargement to 110 mm, and signs of impending rupture. A redo TEVAR was performed using a low-profile device, which was facilitated by a double-guidewire technique due to extreme aortic tortuosity. The postoperative course was favorable.

The patient provided informed consent for all procedures, and the ethics committee at our institute approved the use of anonymized image data for this study.

### 2.2. Definitions

Stent migration was defined, in accordance with current guidelines, as a displacement of the stent >10 mm relative to a primary anatomical landmark [9]. Type III endoleaks are classified into two subtypes: a type IIIA endoleak is a leak between graft components, and a type IIIB endoleak originates from a structural defect within the endograft, such as fabric fracture or tear. Migration-related morbidity was defined as of the following: aortic rupture, type I/III endoleaks (ELs), or aneurysm sac expansion. As proposed by Chen et al. [10], the tortuosity index was used to assess aortic configuration, especially elongation. This is determined by dividing the curved distance along a centerline by the straight spacing between proximal and distal landing zones. In this study, the distance between the most proximal and most distal complete stent circumference was used for the determination of the tortuosity index. Aortic elongation during follow-up was defined as an increase in the centerline-measured aortic length between the left common carotid artery (LCCA) and the celiac trunk (CT).

### 2.3. Image Analysis

**Figure 1:**
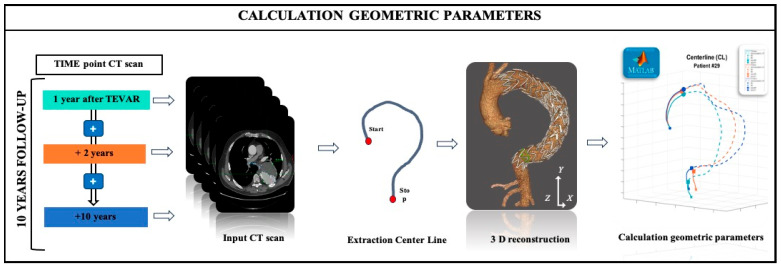
Patient-specific modeling approach used to extract quantitative geometric parameters of the thoracic aorta. Follow-up CT scans at 1, 2, and 10 years post-TEVAR were used to reconstruct the aortic center-line and identify stent graft geometry. The centerlines (green, orange, blue) represent each time point. Segmented line portions indicate the presence of the stent graft.

The key issue in this paper was to quantify the evolution of 3D morphological changes over the course of follow-up from TEVAR, which might be useful for predicting such adverse events as migration of the SG. The analysis was performed using EndoSize software (Therenva, France) [8], a 3D sizing tool optimized for use on a conventional personal computer (Figure 1). Three sets of contrast-enhanced CT images (+1Y, +2Y, and +10Y) were evaluated. All imaging was performed on a 64-slice multidetector scanner (REVO EVO, GE Healthcare, France), with a 0.650 mm in-plane resolution and 3.0 mm slice thickness. A semi-automatic segmentation process, based on three manually selected key points, was used to delineate vascular and bony structures. From this, a 3D aortic centerline (CL) was generated from the sinotubular junction to the celiac trunk. All geometric data were exported as 1 mm spaced centerline coordinates and further analyzed using custom MATLAB (MATLAB_R2021a) tools.

### 2.4. Results

The study parameters included the calculation of the centerline length (L), the angulation (A) and tortuosity (T) along the centerline, and the measurement of the migrated distance of the distal end of the centerline of the thoracic SG. The maximal aortic diameter was assessed perpendicular to the CL, including the aortic thrombus and aortic wall. With this modeling system, we performed the segmentation and centerline extraction process for each time point.

Segmentation was performed for both the native arterial lumen and the SG.

a. Length along the centerline

The centerlines were extracted at three time points. The total length of the SG at +1 year after TEVAR is 208 mm. However, the length of the covered aorta after migration of the distal SG showed a significant increase at two years and ten years of follow-up: 238.0 mm at two years (14.4% increase from 1 year) and 295.0 mm at ten years (23.95% increase from 2 years). Subsequently, the thoracic aorta lengthened progressively since the first intervention: from 388.0 mm at 1 year after TEVAR to 435.0 mm at 2 years (12.11% increase) and to 503.0 mm at 10 years (15.63% increase from 2 years), corresponding to a 30% total increase from 1 year (Table 1).

b. Migration

**Figure 2:**
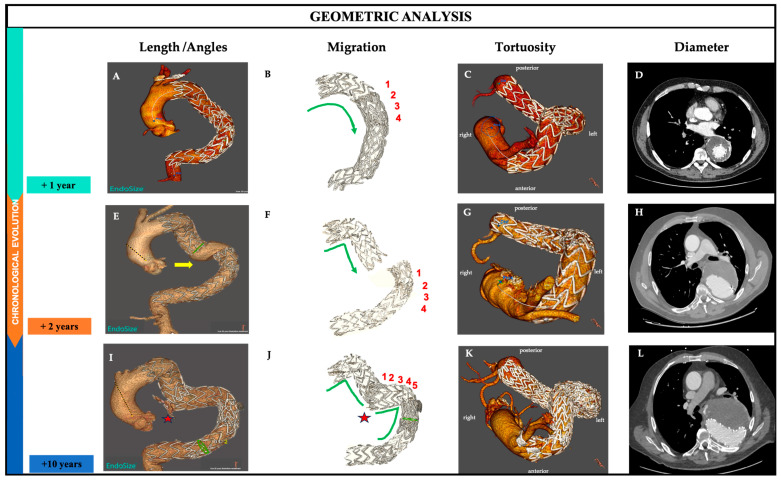
Chronological evolution of aortic morphology and stent-graft (SG) behavior after TEVAR. Three-dimensional reconstructions and axial CT images illustrating morphological changes at 1 year (top, aqua green), 2 years (middle, orange), and 10 years (bottom, blue-violet) post-TEVAR. Each row represents a different time point; each column illustrates a specific geometric dimension: First column (**A**,**E**,**I**): aortic and stent-graft morphology showing length and angulation. Second column (**B**,**F**,**J**): migration and component separation of the stent graft; migrated stents are indicated. Third column (**C**,**G**,**K**): 3D volume rendering showing changes in aortic tortuosity over time. Fourth column (**D**,**H**,**L**): axial CTA slices illustrating sac morphology and maximum aneurysm diameter. At +1 year (**A**–**D**), the sac regressed and the stent-graft configuration remained stable. At +2 years (**E**–**H**), a type IIIa endoleak was detected due to component separation, which was associated with increasing tortuosity and a slight increase in diameter. At +10 years (**I**–**L**), a type IIIb endoleak developed with sac enlargement (up to 110 mm), significant elongation, and pronounced tortuosity. The red star denotes the site of impending rupture. The yellow arrow shows the migration component. This figure illustrates how longitudinal geometric remodeling is closely associated with progressive stent-graft migration and late complications.

We evaluated the overlap length by considering the number of stents. In Figure 2F, we observed four stent displacements, with one additional stent graft inserted at 2 years.

c. Angles

The angulation of the entire endograft and the overall aortic angulation of the whole thoracic aorta were also assessed and calculated between planes perpendicular to the centerline (CL) (Figure 2A,E,I). We observed a gradual increase from 154.73° to 159.94° over 10 years of follow-up for the thoracic aorta, corresponding to an increase of 3%. Meanwhile, the angulation of the stent graft showed a change from 111.23° to 114.4°, reflecting a 2.8% increase (Table 1). This indicates that while there was a slight increase in the angulation of the thoracic aorta and the stent graft, the overall aortic angulation did not significantly change in this patient, suggesting that the healthy sections of the aorta, both proximal and distal, remained stable.

d. Tortuosity

The tortuosity index is calculated by dividing the centerline distance between two zones by the direct distance. We observed (Figure 2B,F,J) an increase from 2.74 to 3.83, corresponding to an increase of nearly 40%, which indicates that the aorta becomes more twisted or curved over time (Table 1). This supports the observed increase in length and the slight change in the “direct/straight” distance between the proximal and distal segments.

e. Diameter

Maximal aortic diameter was assessed perpendicular to the centerline (CL) and included the aortic thrombus and aortic wall (Figure 2D,H,L). It was automatically computed by the post-processing tool and corrected, if necessary, by the reader. The chronological change in the diameters is shown in Table 1: they are 81.6 mm, 74.9 mm, and 100.5 mm (after 1, 2, and 10 years post-TEVAR), corresponding to an increase of 23%.

## 3. Discussion and Literature Review

Stent-graft migration after thoracic endovascular aortic repair (TEVAR) is a multifactorial process with significant clinical consequences. To further support our clinical case that type III endoleaks are a relevant pathogenetic factor in the development of rupture and, in particular, enlargement of the sac and drastic changes in the structure of the stent graft, we conducted an extensive literature search in MEDLINE (via PubMed), Web of Science, and the Cochrane Central Register of Controlled Trials for articles published up to May 2024. No language restrictions were applied, and reference lists of all included studies were manually searched for other potentially eligible studies. Table 2 illustrates critical data on stent migration rates, reintervention rates, comorbidity, and mortality following TEVAR. A significant focus is placed on the role of stent-graft (SG) fatigue over time as a contributor to type III endoleaks, migration, and subsequent changes in the aortic geometry, all of which carry serious clinical implications.

In our literature review, we identified only 11 studies reporting cases of type III endoleaks after TEVAR, for thoracic aortic aneurysms with type III endoleaks in the follow-up (Table 2) [1,11,12,13,14,15,16,17,18,19,20]. Short-term studies, such as those by Jordan et al. [11] and Fairman et al. [20], reported low migration rates of 1% and 2.9%, respectively, over follow-up periods of 1 to 5 years. Similarly, Piffaretti et al. [13] documented a 1.7% migration rate at 12 months, and Kasirajan et al. [12] reported 0.23% over the same period. These findings suggest that stent migration is relatively uncommon in the short term. In contrast, longer-term studies such as those by Beach et al. [1] and Geisbüsch et al. [15] reported migration rates of 11% and 7.3%, respectively, over follow-up periods of up to 10 years. These results underscore the progressive risk of mechanical failure due to stent-graft fatigue over time.

Migration was frequently associated with severe complications. Skrypnik et al. [18] and Yoon & Mell [16] reported very high reintervention rates for migration cases (80% and 100%, respectively). Geisbüsch et al. [15] observed that 44% of migration cases led to complications, with an 11.1% mortality rate. Beach et al. [1] found that migration led to reintervention in 40.9% of cases and complications in 13.6%. These data highlight the clinical importance of early detection and the need for careful long-term follow-up.

Aortic elongation had a significant influence on the occurrence of the late migration of thoracic endografts, thus presenting an independent risk factor for graft migration. Aortic elongation, associated with increased tortuosity, can generate traction forces on the endograft, leading to migration, the loss of landing and overlapping zones, and type III endoleaks and graft separation [21]. This phenomenon is particularly visible in patients with large thoracic aortic aneurysms (TAAs) involving a long aortic segment, as observed in our patient. Morales et al. [22] emphasized that endografts generally conform to the outer curvature of the aorta or aneurysm. Therefore, in cases of aortic expansion or elongation after TEVAR, additional endograft length is necessary. This can lead to the graft being pulled out of the landing or overlapping zones toward the outer curvature, causing type III endoleaks, as seen in our case.

Our results indicate that pronounced tortuosity of the thoracic aortic segment is associated with complications such as endoleaks. Measurements using EndoSize software coupled with MATLAB allowed us to quantify the geometric parameters, revealing that they are key determinants in long-term follow-up, as much as the aortic diameter.

As shown in Table 1, between the first and tenth year post-TEVAR, thoracic aortic length increased from 388 mm to 503 mm, while tortuosity rose from 2.74 to 3.83. In parallel, the endograft length increased (from 208 mm to 295 mm), and its tortuosity rose from 1.31 to 1.84, indicating progressive conformational change and mechanical stress.

Notably, we retrospectively identified that migration signs were already present as early as 24 months postoperatively but were underestimated. Had our current modeling and quantification tools been available and applied at that time, an earlier detection of migration and a preemptive reintervention might have been possible, potentially preventing rupture. This underlines the added value of objective, quantitative follow-up in high-risk anatomies.

These findings support the hypothesis that endograft fatigue plays a central role in late migration. The repetitive biomechanical stress induced by pulsatile blood flow, respiratory motion, and dynamic aortic curvature may lead to gradual deformation and a loss of fixation. Several studies have confirmed that device fatigue and component separation are critical causes of type III endoleaks, a frequent complication of migration [17,18].

Unlike previous reports that offered static snapshots of anatomy, our study offers a longitudinal perspective that highlights the evolving interaction between device and vessel. Prior modeling by Liffman [23] et al. and anatomical studies by Morales et al. [22] and by De Masi et al. [3] support this longitudinal perspective, showing that the outer aortic curvature exerts lateral traction forces on the stent graft, especially in tortuous or elongated segments.

Our results also provide a mechanistic explanation for certain technical choices made by experienced endovascular surgeons—such as the “flowering” deployment technique in tortuous anatomies—which aim to optimize initial SG placement and long-term stability [24]. These intuitive practices may find further justification through geometric modeling. Adequate oversizing and sufficient overlapping distance between multiple stent-graft components are essential technical measures that may help prevent late migration. Furthermore, the influence of stent-graft design and material properties on migration risk should not be overlooked.

Our analysis suggests that geometric remodeling is both a cause and a consequence of stent-graft migration, forming a deleterious feedback loop. This underscores the need for robust surveillance tools capable of detecting early morphological changes before clinical complications arise. EndoSize^®^ [8], in combination with modeling platforms such as MATLAB, offers a valuable method for this purpose. The identification of threshold values for curvature, tortuosity, or length changes may help predict impending migration or failure. Clinically, this approach could refine patient selection, inform deployment strategy, and support timely reintervention. This study has several limitations. While the presented case illustrates a 10-year follow-up, geometric measurements have also been performed on a broader patient group, but with a shorter follow-up duration (3 years) [3]. These findings have not yet been validated across imaging platforms or in multicenter studies. Standardized threshold values for geometric parameters such as elongation or tortuosity remain to be defined for broader clinical application.

## 4. Future Perspectives

Future advances in this field are closely tied to the integration of quantitative imaging tools into precision medicine. Long-term surveillance remains challenging, as patients may develop postoperative complications such as endoleaks, endotension, and stent-graft migration. In this context, the use of post-processing software such as EndoSize^®^ and MATLAB illustrates how semi-automated and quantitative image analysis can provide personalized geometric assessment. These tools may be integrated in the future into automated pipelines supported by artificial intelligence (AI) to identify at-risk anatomical configurations early in follow-up [25]. If the true meaning of translational research in surgery is to transfer knowledge from basic science into techniques that address critical operative needs, then the analytical information extracted from geometric modeling should now be applied systematically to clinical decision-making. Just as computational fluid dynamics (CFD) [26] has demonstrated how flow-related forces are influenced by aortic geometry and may affect device behavior, geometric parameters such as tortuosity, elongation, and angulation can serve as early markers of mechanical instability. Their integration into preoperative planning may help guide endograft sizing, landing zone selection, and the anticipation of long-term complications. AI-based systems could detect subtle geometric changes—including elongation, curvature, or torsion—that precede clinically visible migration. In addition, combining these geometric parameters with patient-specific clinical, biological, and biomechanical data may enable the development of multivariable risk scores or predictive models tailored to individual patient profiles. These approaches, currently under investigation in abdominal aortic aneurysm (AAA) studies, show promise for thoracic pathology as well [27]. The AI-assisted segmentation and automated extraction of relevant indices could assist clinicians in endograft planning, selection, and sizing, thereby optimizing outcomes in complex anatomies.

## 5. Conclusions

This study provides compelling evidence that stent-graft (SG) migration is both a consequence and a driver of progressive aortic geometric remodeling. Migration-induced changes—such as elongation, tortuosity, and angulation—contribute to severe complications, including aneurysm sac re-pressurization, aortic elongation, type III endoleaks, and the risk of rupture. These geometric transformations also increase the complexity of secondary interventions and procedural risks. These observations highlight the importance of early detection and preventive strategies. Our findings support the integration of longitudinal 3D geometric analysis into routine follow-up after TEVAR, as it enables the earlier detection of mechanical instability than standard diameter-based surveillance. Advanced post-processing tools such as EndoSize and MATLAB offer reproducible and quantitative assessments of aortic morphology over time. By combining geometric analysis with individualized therapeutic strategies, clinicians can move toward more precise risk stratification and earlier intervention. This personalized surveillance approach may reduce adverse events and improve the long-term durability of thoracic endovascular repair. The literature further supports the clinical relevance of SG migration, which is strongly linked to fatigue-related mechanical failure, endoleak formation, and high reintervention rates. Migration tends to occur late, underscoring the need for prolonged and standardized imaging surveillance following TEVAR. Future innovation—such as fatigue-resistant alloys, improved anchoring systems, enhanced flexibility, and AI-powered image analysis—may help prevent migration, support better planning, and enable more proactive, personalized, and durable endovascular strategies in complex aortic anatomies.

## Figures and Tables

**Table 1:** Geometrical parameters and migration over time following thoracic endovascular aortic repair (TEVAR).

Geometrical Parameters	+1 Year	+2 Years	+10 Years
**Thoracic Aorta (TA)**			
Length TA (mm)	388.0	435.0	503.0
Angles TA (°)	154.73	159.14	159.94
Tortuosity TA	2.74	3.29	3.83
**Stent Graft (SG)**			
Length SG (mm)	208.0	238.0	295.0
Tortuosity SG	1.31	1.56	1.84
Angles SG (°)	111.23	102.22	114.4
**Migration (n stents)**	0 stents	4 stents	5 stents
**Aneurysm Diameter (mm)**	81.6	74.9	100.5

**Table 2:** Stent-graft migration: incidence, timing, and associated reinterventions, morbidity, and mortality.

Study	Migration Rate, %	Follow-Up, y	Time of Migration, m	Reintervention Rate % n Reintervention/n Migration	Morbidity Rate, % n Morbidity/n Migration	Mortality Rate, % n Deaths/n Migration
Beach et al. [1]	11 (22/200)	0–9.3	29	40.9 (9/23)	13.6 (3/22)	4.5 (1/22)
Jordan et al. [11]	1 (6/66)	1–5	2, 24, 36, 60	0	16.7 (1/6)	0
Kasirajan et al. [12]	0.23 (1/421)	0–1	12	ND	ND	ND
Piffaretti et al. [13]	1.7 (2/117)	0–12	12	16.7 (2/12)	16.7 (2/12)	0
Foley et al. [14]	3.1 (6/195)	0–5	12, 24, 36, 48, 60	16.7 (1/6)	16.7 (1/6)	0
Geisbüsch et al. [15]	7.3 (9/123)	0.5–10	8, 11, 14, 22, 30, 32, 43, 49, 64	55.5 (5/9)	44.4 (4/9)	11.1 (1/9)
Yoon and Mell [16]	1.6 (1/63)	4.8	14	100 (1/1)	100 (1/1)	0
Matsumura et al. [17]	7.6 (12/158)	0–5	3.2 ± 1.7 years	16.7 (2/12)	16.7 (2/12)	0
Skrypnik et al. [18]	2.6 (5/194)	0.5–10	12, 19, 31, 43, 64	80 (4/5)	100 (5/5)	40 (2/5)
Torsello et al. [19]	0.8 (1/127)	0–1	12	100 (1/1)	100 (1/1)	0
Fairman et al. [20]	2.9 (3/105)	0–1	12, 24, 60	0	0	0

Abbreviation: n: number; ND: no date; y: years; m: months.
